# Isolation and Phylogenetic Analysis of Reemerging Pseudorabies Virus Within Pig Populations in Central China During 2012 to 2019

**DOI:** 10.3389/fvets.2021.764982

**Published:** 2021-11-16

**Authors:** Hui-Hua Zheng, Yi-Lin Bai, Tong Xu, Lan-Lan Zheng, Xin-Sheng Li, Hong-Ying Chen, Zhen-Ya Wang

**Affiliations:** ^1^Zhengzhou Major Pig Disease Prevention and Control Laboratory, College of Veterinary Medicine, Henan Agricultural University, Zhengzhou, China; ^2^College of Veterinary Medicine, Northwest A&F University, Xianyang, China; ^3^Key Laboratory of “Runliang” Antiviral Medicines Research and Development, Institute of Drug Discovery & Development, Zhengzhou University, Zhengzhou, China

**Keywords:** pseudorabies virus, sequencing, phylogenetic analysis, reemerging virus, pig disease

## Abstract

To understand the biological characteristics of the reemerging pseudorabies virus (PRV) strains, a total of 392 tissue samples were collected from diseased pigs during reemerging PR outbreaks between 2012 and 2019 on farms in central China where swine had been immunized with Bartha-K61 and 51 (13. 01%) were positive for the gE gene by PCR. Sixteen PRV strains were isolated and caused clinical symptoms and death in mice. Subsequently, gE, gC, gB, and gD complete genes were amplified from the 16 PRV isolates and sequenced. Phylogenetic analysis based on these four gene sequences shows that the 16 PRV isolates were more closely related to the Chinese PRV variants (after 2012) but genetically differed from early Chinese PRV isolates (before 2012). Sequence analysis reveals that PRV isolates exhibited amino acid insertions, substitutions, or deletions compared with early Chinese PRV isolates and European–American PRV strains. In addition, this is the first report that eight isolates (8/16) in this study harbor a unique amino acid substitution at position 280 (F to L) of the gC protein, and six isolates have an amino acid substitution at position 338 (A to V) of the gD protein compared with the Chinese PRV variants. The emulsion containing inactivated PRV NY isolate could provide complete protection against the NY isolate. This study might enrich our understanding of the evolution of reemerging PRV strains as well as pave the way for finding a model virus to develop a novel vaccine based on reemerging PRV strains.

## Introduction

Pseudorabies (PR), or Aujeszky's disease, is an economically important viral disease of swine worldwide and poses a great threat to the pork industry (1, 2). Infected pigs display a range of symptoms, including reproductive failure in sows, severe respiratory disease in adult pigs, fatal neurological disorders, and high mortality in newborn piglets (3). The first description of PR was made in America as early as 1813, and the first recorded case in China was in 1948 with a following epidemic in pigs in the late 1980s (4). Due to the use of the gE-negative vaccine strain Bartha-K61 imported from Hungary, PR was well-controlled from the 1990s to 2010 in China (5, 6). Since late 2011, however, reemerging PR outbreaks have occurred among Bartha-K61-immunized swine herds on many Chinese farms and led to tremendous economic losses (6).

The causative agent responsible for PR is the pseudorabies virus (PRV), which is a member of the family *Herpesviridae*, subfamily *Alphaherpesvirinae*, and genus *Varicellovirus*. The genome of PRV is a linear double-stranded DNA that encodes more than 70 proteins (7). Among these proteins, glycoproteins B (gB), C (gC), and D (gD) induce cellular and humoral immune responses and are related to the process of virus fusion (8–11). Furthermore, the gC gene is frequently used to analyze the evolutionary relationships of PRV, and the attachment of the virus to cells is initiated by the binding of the gC protein to heparan sulfate proteoglycans (12–14). The glycoprotein E (gE) is a major virulence determinant of PRV but is not essential for virus replication (15). In light of this fact, gE-deleted vaccines (Bartha-K61 vaccine) were developed, and the vaccines, together with a corresponding serological test to detect antibodies against the gE protein, have played a key role in the program of the elimination of PR.

Some research indicates that the causative agent of currently circulating PRV is confirmed to be novel PRV strains (PRV variants), which are genetically different from the early Chinese PRV strains, and the Bartha-K61 vaccine did not provide full protection against the PRV variants due to enhanced pathogenicity and genetic differentiation of PRV variants (16, 17). Furthermore, Sun's study shows that reemerging PRV strains spread widely to many provinces of China between 2012 and 2017, and other research also reveals that the gE, gC, gB, and gD amino acid (aa) sequences of reemerging PRV strains include alterations (3, 13, 18–20). For further investigating the molecular epidemiology of reemerging PRV strains, this study reports the detection and genetic analysis of PRV from pigs in central China between 2012 and 2019 and is also evaluated to find a candidate virus for the development of novel and efficient PR vaccines.

## Materials and Methods

### Cells, Virus, and Sample Collection

Swine testicle (ST) cells and PRV strain Min-A were purchased from the China Institute of Veterinary Drug Control, Beijing, China, and the ST cells were passaged in Dulbecco's modified Eagle's medium (DMEM) (Gibco, Carlsbad, CA, USA) supplemented with 10% heat-inactivated fetal bovine serum (Gibco).

From 2012 to 2019, a total of 392 tissue samples (including lungs, brains, kidneys, lymph nodes, and spleens) were collected from 392 diseased pigs during reemerging PR outbreaks on 39 farms in central China (including in Kaifeng, Shangqiu, Zhoukou, Luoyang, Sanmenxia, Nanyang, Zhumadian, Xinyang, Anyang, Xinxiang, Jiaozuo, Puyang, Hebi, Zhengzhou, Pingdingshan, Xuchang, and Luohe cities) where swine had been immunized with Bartha-K61, and the sample numbers per year are 80, 50, 60, 56, 32, 36, 38, and 40, respectively.

### PCR Detection

Viral DNA was extracted from 200 μL supernatants of the suspension of tissue using the DNA Miniprep Kit (Omega, Norcross, Georgia, USA) according to the manufacturer's instructions and subsequently screened for the presence of the PRV genome by PCR. A pair of primers gEp-F/R (gEp-F: 5′-TGGGACACGTTCGACCTGATG-3′, gEp-R: 5′-CCTTGATGACCGTGACGTA CT-3′) were designed from the reference strain PRV Ea (accession number AF171937) to amplify the partial gE gene with the length of 429 bp. PCR was performed in a 25-μL volume mixture consisting of 12.5 μL Premix *Taq* (Takara, Dalian, China), 3 μL extracted DNA template, 7.5 μL of ddH_2_O, 1 μL DMSO, and 0.5 μL each of primer (50 μM), and the cycling protocol was an initial denaturation at 95°C for 5 min, followed by 35 cycles at 95°C for 30 s, 53°C for 30 s, and 72°C for 1 min with a final step of 72°C for 10 min. The PCR amplification using the DNA of PRV strain Min-A was considered as a positive control, and the product with ddH_2_O instead of the DNA template was used as the negative control. The PCR product was visualized by electrophoresis in a 1.5% agarose gel containing ethidium bromide under ultraviolet light.

### Virus Isolation and Identification

For virus isolation, homogenate supernatants of positive tissue samples confirmed by PCR were filtered using a 0.22-μm filter (EMD Millipore, Billerica, MA, USA) and inoculated into ST cells. The inoculated cells were observed daily. When 80% of cells showed the cytopathic effects (CPEs), viruses were harvested by three freeze–thaw cycles and were further plaque purified. Subsequently, the virus was corroborated by PCR with primers gEp-F/R.

### Pathogenic Test in Mice

One hundred seventy six-week-old healthy BALB/c female mice were randomly divided into 17 groups of 10. Mice in PRV-injected groups were inoculated subcutaneously (s.c.) with the different isolates of a virus titer of 10^5.0^ 50% tissue culture infectious doses (TCID_50_)/dose as previously described, respectively (21). Mice in the DMEM-injected group were inoculated with the same dose of DMEM as control. After the inoculation, the mice were monitored daily for clinical signs for 7 days.

### Physicochemical Properties of Virus

Physicochemical properties of NY isolate as a representative PRV were assayed as described previously (22). Briefly, the 500 μL cell culture medium–infected NY isolate was added into eight 1.5-mL Eppendorf tubes. Tubes 1 and 2 were treated by a water bath at 56°C for 1 h and chloroform at 4°C for 30 min, respectively. Tubes 3 and 4 were treated by adjusting the culture medium pH to 3.0 and 11.0 with 0.1 M HCl/NaOH solutions. After incubation at 37°C for 1 h, the pH values were then adjusted back to 7.0 by 0.1 M NaOH solutions. Tube 5 was digested with trypsin in a 37°C water bath for 1.5 h and then added 4 mL of the inactivated fetal bovine serum to terminate the reaction. Tube 6 was incubated with formaldehyde at 37°C for 2 d. Tube 7 was treated under ultraviolet rays for 30 min. Tube 8 was used as a negative control. Viruses of the eight tubes were inoculated on ST monolayers, respectively, and TCID_50_ was determined by the Reed–Muench method, respectively (23). In addition, the seventh passage of the NY strain with propagating in ST cells was stained with uranyl acetate and examined using a Hitachi TEM transmission electron microscope (Hitachi, Japan).

### Sequencing and Phylogenetic Analysis

The complete gE, gC, and gB genes of PRV were amplified from viral DNA extracted from PRV isolates using specific primers gE-F/R, gC-F/R, and gB-F/R designed in our laboratory (21). The complete gD gene was amplified by PCR from viral DNA using a pair of primers gD-F/R (gD-F: 5′-ATCCACTCCCAGCGGTCCACAAAAT−3′, gD-R: 5′- AAAAACAGCAGCGTCCCGTCTATCG−3′) with the same PCR volume mixture as that of the partial gE gene, and the cycling protocol was as follows: an initial denaturation at 95°C for 5 min, 35 cycles of denaturation at 95°C for 55 s, annealing at 57°C for 1 min, extension at 72°C for 100 s, and a final extension at 72°C for 10 min. Then, the genes were ligated with the vector pMD18-T (Takara) and separately introduced to *Escherichia coli* DH-5α cells (Takara) by transformation according to the manufacturer's instructions. The positive recombinant plasmids carrying gE, gC, gB, and gD genes were sent to Sangon Biotech Shanghai Co., Ltd., for DNA sequencing, and all sequencing reactions were performed in duplicate.

Phylogenetic trees were constructed based on the complete gE, gC, gB, and gD genes of PRV isolates and reference strains available in GenBank using MEGA software, version 7.0 (www.megasoftware.net) by the neighbor-joining (NJ) method with 1,000 bootstrap replicates (24). Evolutionary distances were computed by the pairwise distance method with the maximum composite likelihood model. Sequences of PRV strains listed in Table 1 retrieved from NCBI were used as references.

**Table 1:** The information of PRV strains for sequence alignment and phylogenetic analysis.

**Study virus**	**Accession number**	**Origin**	**Reference virus**	**Accession number**	**Origin**
**JY**	KF017615^E^/KF997104^C^/KX880453^B^/KX880468^D^	Henan, China, 2012	HN2012	KP722022 ^G^	China, 2012
**MZ1**	KF130881/KF997097/KX880456/KX880471	Henan, China, 2012	HNX	KM189912	China, 2012
**MZ2**	KF130882/KF997099/KX880457/KX880472	Henan, China, 2012	HNB	KM189914	China, 2012
**LGX**	KF042384/KF997098/KX880454/KX880469	Henan, China, 2012	TJ	KJ789182	China, 2012
**NY**	KF130883/KF997096/KX880458/KX880473	Henan, China, 2012	JS-2012	KP257591	China, 2012
**ZK**	KF130886/KF997100/KX880464/KX880479	Henan, China, 2012	ZJ01	KM061380	China, 2012
**ZM**	KF130887/KF997102/KX880465/KX880480	Henan, China, 2012	SC	KT809429	China, 1986
**M5**	KF130880/KF997095/KX880455/–	Henan, China, 2012	Ea	KU315430	China, 1990
**GY**	KF042383/KF997103/KX880452/–	Henan, China, 2012	LA	KU552118	China, 1997
**SMX**	KP192494/KR025920/KX880459/KX880474	Henan, China, 2014	Fa	AF403049 ^E^/AF403051 ^C^/–^B^/AY196984^D^	China, 1993
**WZ**	KR119070/KR119071/KX880461/KX880476	Henan, China, 2014	Hercules	KT983810	Greece, 2010
**YZ**	KP192495/KR153193/KX880463/KX880478	Henan, China, 2014	Kaplan	JF797218	Hungary, 1959
**BP**	KP318116/KP318117/KX880451/KX880466	Henan, China, 2014	Becker	JF797219	America, 1967
**WY**	KF130884/KF997094/KX880460/KX880475	Henan, China, 2013	Kolchis	KT983811	Greece, 2010
**YY**	KF130885/KF997101/KX880462/KP259814	Henan, China, 2013	Bartha	JF797217	Hungary, 1960
**XC**	MW2388925/MW073286/MW654204/–	Henan, China, 2018			

E *Represents gE genes of PRV strains*.

C *Represents gC genes of PRV strains*.

B *Represents gB genes of PRV strains*.

D *Represents gD genes of PRV strains*.

G *Represents whole genomes of PRV reference strains. The 16 strains sequenced in this study are shown in bold*.

### Immunogenicity Test

The representative PRV NY isolate was inoculated into ST cells. The cell culture suspension of virus titer of 10^5.0^ TCID_50_ was inactivated by incubating with moderate formalin (Sigma-Aldrich) at 37°C for 24 h and then emulsified with Freund's complete adjuvant and Freund's incomplete adjuvant, respectively. Sixty six-week-old healthy BALB/c female mice were randomly divided into four groups of 15 mice each. Mice in Group 1 were injected with 500 μL inactivated NY isolate containing Freund's complete adjuvant in the first week, followed by injecting with an equal volume of emulsifier with Freund's incomplete adjuvant in the third week, and finally with the inactivated virus in the fifth week. Groups 2–4 were injected with the same dose of Bartha-K61, Hubei 98 (the PRV vaccine strain with deleting three important virulence factors) or DMEM in the first, third, and fifth weeks, respectively. On the 32nd day after the final injection, blood samples were collected from five mice of each group for the serum neutralization assay, and the remaining mice of each group were challenged with 10^5.0^ TCID_50_ PRV NY isolate. Serum-neutralizing antibodies against PRV were detected as described previously (25).

## Results and Discussion

Of the 392 samples, 51 were positive for the PRV gene, yielding an average positive rate of 13.01% (51/392), which was coincident with the reports of the prevalence of novel PR in China (6, 19, 26, 27). Besides this, the positive rates of PRV detection from 2012 to 2019 were 17.5% (14/80), 20.0% (10/50), 25.0% (15/60), 14.29% (8/56), 12.5% (4/32), 11.11% (4/36), 7.89% (3/38), and 6.61% (8/40), respectively.

**Figure 1:**
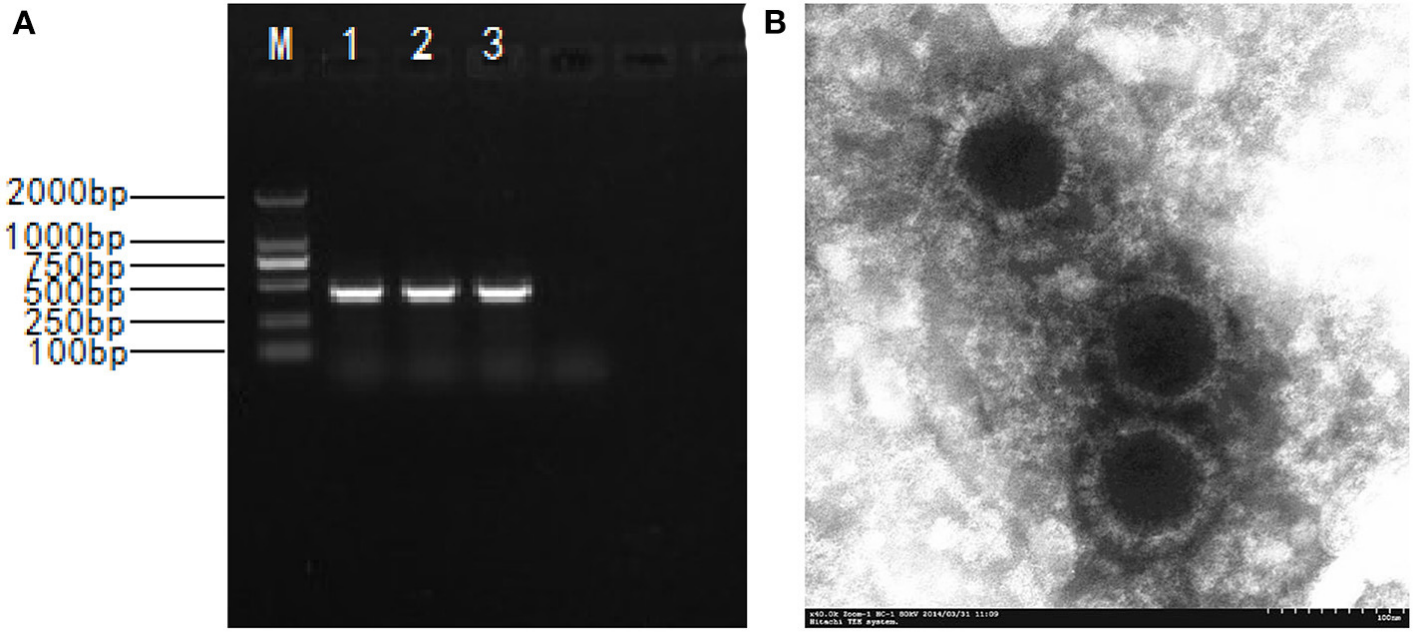
The isolation and identification of the PRV. **(A)** Amplified PCR product of 429 bp; M. DNA marker DL2000; 1-3. PCR products; 4. Negative control; **(B)** Electronograph of PRV NY strain.

PRV gE gene–positive tissue samples were inoculated into ST cells, and distinct CPEs characterized by cell rounding, pyknosis, and degeneration were observed after three blind passages on ST cells. The expected products of 429 bp (Figure 1A) were amplified from infected cells with the specific primers of the gE gene. Sixteen PRV isolates were obtained and formally named NY, GY, LGX, MZ1, MZ2, ZM, ZK, JY, M5, YY, WY, BP, YZ, SMX, WZ, and XC (Table 1). TCID_50_ of these PRV isolates were during 10^5.375^ to 10^9.0^/0.1 mL with the highest TCID_50_ of NY and the lowest of XC. The 16 isolates were highly pathogenic in mice and caused skin inflammation, neural symptoms, and death in all experimentally infected mice 48–72 h after challenge. In contrast, all mice of the negative control group survived, which is in agreement with previous studies (21, 28).

The physicochemical property results of the representative PRV NY isolate are shown in Table 2. The NY isolate was sensitive to chloroform, trypsin, formaldehyde, and ultraviolet ray, demonstrating that it belonged to the enveloped virus (2). The virus was not inactivated until the heating time was above an hour at 56°C, showing that the NY isolate had high heat resistance. When the culture medium pH was adjusted to 3.0 or 11.0, no detectable titers were observed. In the ST cells infected with NY isolate, a circular viral particle of about 110~150 nm was observed, and virus particles exhibited envelope protein with a radially arranged spike (Figure 1B), which is basically consistent with the morphological features of PRV. These results are identical to the physicochemical properties of PRV (29).

**Table 2:** Detection results of physicochemical property tests for PRV NY isolate.

**Treatment**	**Test group (TCID_**50**_/0.1 mL)**	**Control group (TCID_**50**_/0.1 mL)**	**Difference**
Heat resistance	10^1^	10^9.0^	8.0
Chloroform resistance	10^0.75^	10^9.0^	8.25
Acid resistance	10^0^	10^9.0^	9.0
Alkali resistance	10^0^	10^9.0^	9.0
Trypsin resistance	10^0^	10^9.0^	9.0
Formaldehyde resistance	10^1.1^	10^9.0^	7.9
Uv resistance	10^8.0^	10^9.0^	1.0

**Figure 2:**
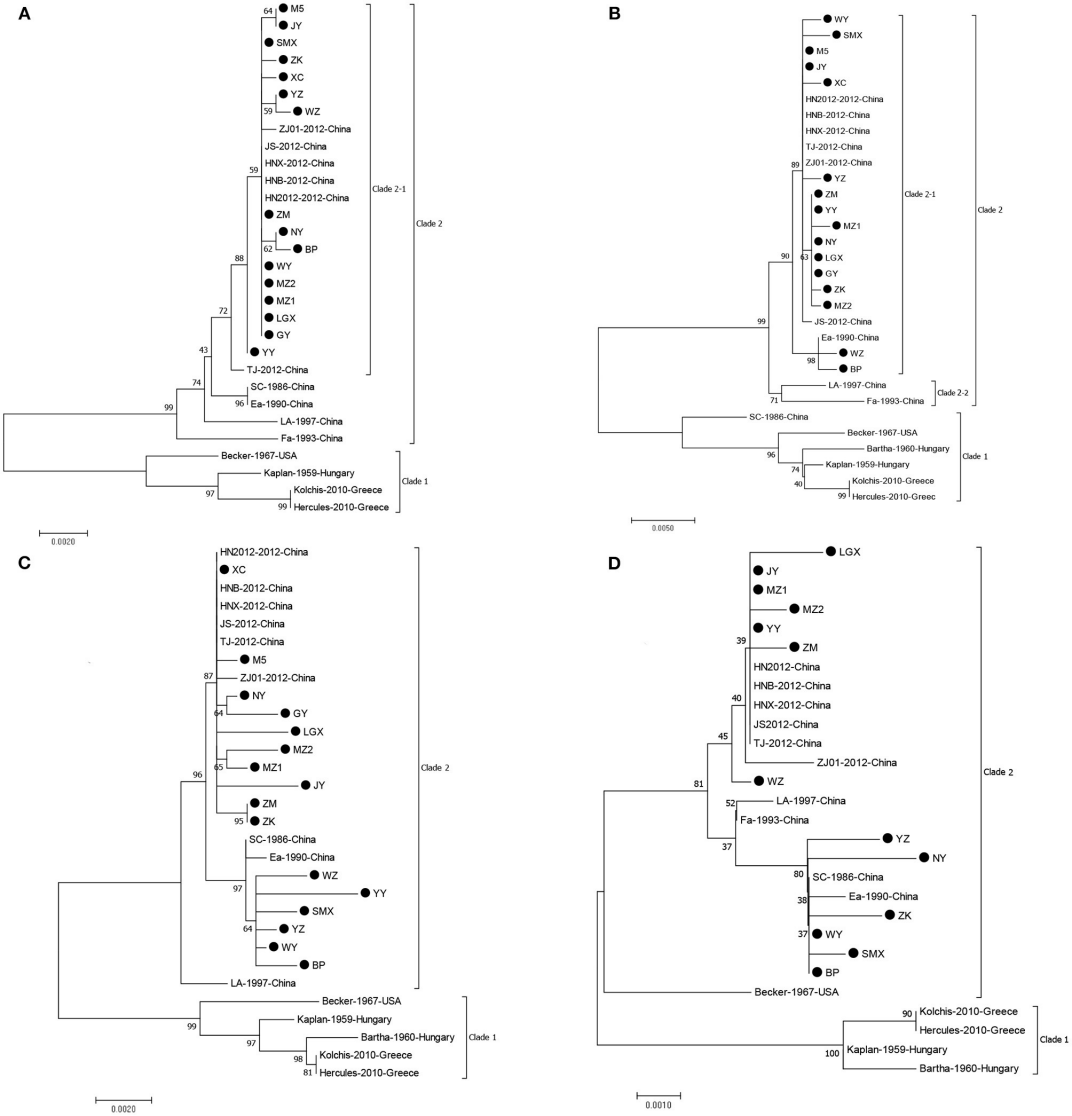
Phylogenetic tree constructed by aligning nucleotide sequences of gE **(A)**, gC **(B)**, gB **(C)**, and gD **(D)** genes of the PRV isolates and reference strains using the NJ method. Bootstrapping with 1,000 replicates was performed to determine the percentage reliability for each internal node. Black circles indicate PRV field isolates in this study.

We successfully obtained the gE, gC, gB, and gD genes of 16 PRV isolates with 1,867, 1,587, 2,867, and 1,494 bp and submitted the sequences to GenBank under the accession numbers listed in Table 1. Sequencing analysis of the PRV gE, gC, gB, and gD genes reveals maximal aa (nucleotide) sequence divergences of 0.3% (0.2%), 1.7% (0.7%), 2.3% (0.9%), and 1.3% (0.8%) within the 16 isolates and 4.3% (2.3%), 7.9% (4.5%), 4.4% (2.1%), and 3.3% (1.5%) compared with European–American PRV strains, respectively. The maximal aa (nucleotide) sequence divergence of the four genes of the 16 isolates were 2.3% (0.9%), 6.1% (3.5%), 2.0% (0.8%), and 1.0% (0.7%) compared with those strains prevalent in China before 2012 and were 0.9% (0.3%), 1.9% (0.5%), 1.5% (0.7%), and 1.3% (0.8%) after 2012. On the basis of NJ trees of the gE, gC, gB, and gD genes derived from the 16 isolates and PRV reference strains, four trees (Figure 2) could be divided into two main clades, Clades 1 and 2. All 16 isolates in this study belong to Clade 2, together with the Chinese PRV strains, and most of them based on the gE and gC genes are clustered in Clade 2-1 along with the six Chinese PRV variants (after 2012), which are similar to the PRV variants (19). Clade 1 is composed of the remaining strains (European–American PRV strains). These findings reveal that the 16 isolates are genetically closer to the PRV variants but differ from early Chinese PRV isolates. Interestingly, the 16 isolates are located in different clades for four genes. For gC, gB, and gD genes, isolate BP is in the same small branch as one early Chinese PRV Ea strain (Figures 2B–D), whereas the BP isolate belongs to Clade 2-1, including the six Chinese variant PRV strains (Figure 2A), suggesting that interclade recombination events might happen among the PRV strains.

In the deduced gE aa sequences, compared with all strains of Clade 1 (Figure 2A), two aa insertions at positions 48 (D) and 497 (D) were found in the strains of Clade 2-1, and these were not observed for all other strains. Furthermore, all the 16 isolates of Clade 2-1 had 20 aa interspersed substitutions (at positions 54, 59, 63, 106, 121, 149, 179, 181, 215, 216, 449, 472, 474, 504, 509, 512, 522, 526, 577, and 578) except for the YY isolate at position 512 and BP isolate at position 577 (Supplementary Table 1). In comparison with the early Chinese PRV strains, all isolates except for the YY isolate had two aa substitutions at positions 449 (V to I) and 512 (G to S). Compared with the variants, there was an aa substitution at position 386 (T to M) for both isolates NY and BP, and two aa substitutions at positions 329 (W to R) and 532 (S to G) were shared for both WZ and ZK isolates. The gE protein is the major virulent protein of PRV, and only a few aa changes could transform the virulence of the virus in a previous study (30). Hence, these aa changes might also influence the virulence of reemerging PRV strains in China.

Compared with gC proteins of all strains of Clade 1 (Figure 2B), seven aa insertions at positions 63–69 (AAASTPA) of the gC protein were found in the strains of Clade 2, and the strains of Clade 2-1 (except for strain Ea) had 24 aa interspersed substitutions at positions 16, 25, 52, 55, 57, 59, 60, 61, 87, 90, 102, 130, 142, 187, 240, 431, 437, 449, 457, 461, 467, 485, 486, and 487 (Supplementary Table 2). As the important neutralizing antigen of PRV, gC protein is the main virulent protein, which conducts the adsorption process between virus and target cells (30). Therefore, these changes might alter the structure of gC glycoprotein and then affect the adhesion of the virus to host cells. Interestingly, eight strains, MZ1, NY, LGX, MZ1, XY, ZK, ZM, and GY, in this study harbor an additional aa substitution at position 280 (F to L) compared with reference strains, which is the first report that the characteristic aa substitution (at position 280) exists in the Chinese variant PRV strains' gC.

For the gB protein of the principle immunogen of the virus, alignments of the gB protein show that 16 PRV isolates in this study exist in the insertions, deletions, and substitutions of aa compared with the Bartha strain (Supplementary Table 3) and deletion of three aas (S, P, and G) at positions 75, 76, and 77 by comparison with the strains of Clade 1 (Figure 2C). These deletions of three aas and some aa changes are in agreement with previous studies (14, 19). The BP, SMX, WY, WZ, YY, and YZ have four aa changes at positions 85 (A to T), 454 (R to K), 563 (H to Q), and 740 (T to A) compared with the Chinese variants, and the remaining isolates have an aa change at position 454 (R to K) in comparison with the early Chinese strains. In addition, there were unique aa substitutions of PRV isolates, such as seven aa interspersed substitutions for JY; five aa changes for YY and LGX; four aa changes for MZ2, WZ, and GY; three aa changes for MZ1 and SMX; two aa changes for ZK, ZM, and M5; and one aa change for NY, WY, and YZ. These aa changes might result in the alteration of the neutralizing epitope of the gB protein and consequently loss of the protective efficacy of the previous vaccine Bartha-K61 in China.

Compared with the gD proteins of all strains of Clade 1 (except for the Becker) (Figure 2D), the isolates from this study had 10 aa interspersed substitutions at positions 207, 209, 212, 280, 281, 288, 309, 342, 346, and 395 (Supplementary Table 4). Compared with the variants, the NY isolate has three aa changes at positions 384 (K to E), 395 (A to T), and 401 (Q to L), and the YZ isolate has two aa insertions (R and P) at positions 278 and 279 and one aa substitution at position 323 (P to L), respectively. In addition, the ZK isolate harbors two aa substitutions at positions 143 (F to L) and 170 (V to F), and SMX, MZ2, LGX, and ZM have an aa substitution at positions 117 (C to S), 242 (G to D), 331 (P to L), and 353 (R to C), respectively. Remarkably, in six strains, BP, NY, SMX, WY, YZ, and ZK, an additional aa substitution was identified at position 338 (A to V) compared with the Chinese variants, which first reported that the characteristic aa substitution (at position 338) existed in the Chinese variant PRV strains' gD. For PRV, the gD protein is reported to play a role in attachment and affect the infectivity of the virus (30). These aa changes of the gD protein, therefore, might be the reason for the alteration of the virulence of reemerging PRV strains in China.

All mice in the four groups did not display adverse reactions after vaccination (data not shown). After the challenge with the PRV NY isolate, mice in group 1 survived without typical PR symptoms, and the mortality was 0% (0/10). The mortalities of groups 2–4 were 50% (5/10), 30% (3/10), and 100% (10/10), and typical PR symptoms appeared, such as depression, itching, and scratching. As for neutralizing antibodies against PRV, mice in groups 1–3 induced neutralizing antibodies with titers of 1:82, 1:24, and 1:22, respectively. No neutralizing antibodies were detected in group 4 as control. The neutralization titer between the PRV NY isolate and its serum was the highest, followed by the Bartha-K61 and Hubei 98 strains, which indicate that the PRV NY strain might have cross-antigenicity with both the Bartha-K61 and Hubei 98 strains with the different antigenicity. These results agree with previous findings (6), further indicating that the Bartha-K61 vaccine cannot provide full protection against the reemerging PRV strains. Therefore, the development of novel vaccines based on the reemerging PRV strains is urgent.

Live vaccines are currently employed to control PR on many swine farms in China, mainly based on strain Bartha-K61, which is an attenuated strain of PRV produced by extensive *in vitro* passages and has a well-characterized deletion of the complete gE and partial gI genes encoding proteins that attenuate virulence, and Bartha-K61 has played a key role in the eradication of PR (31). From 2005 to 2010, the positive PRV gE antibodies were detected in only 3–5% of serum samples (32). Since 2012, severe PRV outbreaks have occurred on several swine farms and spread rapidly to most of China (6, 20, 27, 32). Although the PRV infection has recently decreased since the Chinese government proposed the eradication program based on PR in 2011, it remains not completely eradicated (14, 19, 20). Some research demonstrates that novel PR is caused by reemerging PRV strains (6, 13, 19, 20, 33), and this study further confirms reemerging PRV strains are prevalent in China. To control and prevent the PRV infection in swine herds, we should develop effective vaccines and combine them with other integrated control measures, such as serological and virological monitoring, and biosafety procedures. In addition, the Chinese government should call on all relevant practitioners (farmers, veterinarians, scientists, vaccine manufacturers, officials, and communities) to join hands for fighting the disease and learn from many countries in South America, North America, and Europe that successfully eliminated PR for a long time. Facing the prevalence of PR, it is worth further study to evaluate whether the current immunization procedures and biosafety measures are reasonable and effective.

In conclusion, this study suggests that PR is not yet eradicated and still exists in central China, and the aas of gE, gC, gB, and gD of 16 PRV isolates from this study show mutations compared with the early Chinese PRV strains. Further, it can be speculated that these variations may be the cause of the reemergence of PR in China. The question of whether the genetic variations seen in complete gE, gC, gB, and gD genes of these PRV isolates affect the pathogenicity of PRV is currently under further investigation, and the complete genome sequencing of the isolates is also necessary.

## Data Availability Statement

The datasets presented in this study are deposited in the online repositories. The names of the repository/repositories and accession number(s) can be found in the article/Supplementary Material.

## Ethics Statement

All experimental procedures were reviewed and approved by the Henan Agriculture University Animal Care and Use Committee (license number SCXK (Henan) 2013-0001).

## Author Contributions

H-HZ and H-YC have made substantial contributions to the conception, design of the work, the acquisition, and analysis. TX and Z-YW carried out interpretation of data. X-SL and L-LZ discussed and prepared the final report. H-HZ, H-YC, and Y-LB have drafted the work or substantively revised it. All authors have read and approved the final manuscript.

## Funding

This work was supported by the Zhongyuan High Level Talents Special Support Plan (No. 204200510015), Program for Scientific and Technological Innovation Talents in Universities of Ministry of Education of Henan Province (No. 21HASTIT039), and Fang's family (Hong Kong) foundation.

## Conflict of Interest

The authors declare that the research was conducted in the absence of any commercial or financial relationships that could be construed as a potential conflict of interest.

## Publisher's Note

All claims expressed in this article are solely those of the authors and do not necessarily represent those of their affiliated organizations, or those of the publisher, the editors and the reviewers. Any product that may be evaluated in this article, or claim that may be made by its manufacturer, is not guaranteed or endorsed by the publisher.
