# Supplementary material for: Isolation and Phylogenetic Analysis of Reemerging Pseudorabies Virus Within Pig Populations in Central China During 2012 to 2019
Source: Front Vet Sci. 2021 Nov 16;8:764982. doi: 10.3389/fvets.2021.764982 (PMC8635136; doi:10.3389/fvets.2021.764982)
Supplement: Supplementary file 1 [file Table_1.DOCX]

Supplementary Table 1 Amino acid (AA) mutations of gE protein of 16 PRV isolates from this study and 13 PRV reference strains compared with Becker strain

| Strain | Amino acid point mutation positions (position of alignment) | | | | | | | | | | | | |
| --- | --- | --- | --- | --- | --- | --- | --- | --- | --- | --- | --- | --- | --- |
|  | 48 | 54 | 59 | 63 | 69 | 75 | 80 | 90 | 106 | 122 | 125 | 149 | 163 |
| Becker | — | G | D | N | A | L | E | S | V | A | M | R | Q |
| BP | D | D | N | D | · | · | · | · | L | S | T | M | R |
| GY | D | D | N | D | · | · | · | · | L | S | T | M | R |
| JY | D | D | N | D | · | · | · | · | L | S | T | M | R |
| LGX | D | D | N | D | · | · | · | · | L | S | T | M | R |
| M5 | D | D | N | D | · | · | · | · | L | S | T | M | R |
| MZ1 | D | D | N | D | · | · | · | · | L | S | T | M | R |
| MZ2 | D | D | N | D | · | · | · | · | L | S | T | M | R |
| NY | D | D | N | D | · | · | · | · | L | S | T | M | R |
| SMX | D | D | N | D | · | · | · | · | L | S | T | M | R |
| WY | D | D | N | D | · | · | · | · | L | S | T | M | R |
| WZ | D | D | N | D | · | · | · | · | L | S | T | M | R |
| YY | D | D | N | D | · | · | · | · | L | S | T | M | R |
| YZ | D | D | N | D | · | · | · | · | L | S | T | M | R |
| ZK | D | D | N | D | · | · | · | · | L | S | T | M | R |
| ZM | D | D | N | D | · | · | · | · | L | S | T | M | R |
| XC | D | D | N | D | · | · | · | · | L | S | T | M | R |
| HN2012 | D | D | N | D | · | · | · | · | L | S | T | M | R |
| HNX | D | D | N | D | · | · | · | · | L | S | T | M | R |
| HNB | D | D | N | D | · | · | · | · | L | S | T | M | R |
| TJ | D | D | N | D | · | · | · | · | L | · | T | M | R |
| ZJ01 | D | D | N | D | · | · | · | · | L | S | T | M | R |
| JS-2012 | D | D | N | D | · | · | · | · | L | S | T | M | R |
| LA | D | D | N | D | · | · | · | · | L | S | T | M | R |
| Fa | · | · | N | D | T | · | V | P | L | S | T | M | R |
| Ea | D | · | N | D | · | · | · | · | L | S | T | M | R |
| SC | D | · | N | D | · | · | · | · | L | S | T | M | R |
| Hercules | · | · | · | · | · | R | · | · | · | · | T | · | R |
| Kaplan | · | · | · | · | · | R | · | · | · | · | T | · | R |
| Kolchis | · | · | · | · | · | · | · | · | · | · | T | · | R |

Continue

| Strain | Amino acid point mutation positions (position of alignment) | | | | | | | | | | | | |
| --- | --- | --- | --- | --- | --- | --- | --- | --- | --- | --- | --- | --- | --- |
|  | 216 | 300 | 309 | 329 | 348 | 386 | 396 | 404 | 448 | 449 | 472 | 474 | 497 |
| Becker | A | A | —— | W | A | T | W | A | A | V | G | R | —— |
| BP | D | · | · | · | · | M | · | · | · | I | R | H | D |
| GY | D | · | · | · | · | · | · | · | · | I | R | H | D |
| JY | D | · | · | · | · | · | · | · | · | I | R | H | D |
| LGX | D | · | · | · | · | · | · | · | · | I | R | H | D |
| M5 | D | · | · | · | · | · | · | · | · | I | R | H | D |
| MZ1 | D | · | · | · | · | · | · | · | · | I | R | H | D |
| MZ2 | D | · | · | · | · | · | · | · | · | I | R | H | D |
| NY | D | · | · | · | · | M | · | · | · | I | R | H | D |
| SMX | D | · | · | · | · | · | · | · | · | I | R | H | D |
| WY | D | · | · | · | · | · | · | · | · | I | R | H | D |
| WZ | D | · | · | R | · | · | · | · | · | I | R | H | D |
| YY | D | · | · | · | · | · | · | · | · | I | R | H | D |
| YZ | D | · | · | · | · | · | · | · | · | I | R | H | D |
| ZK | D | · | · | · | · | · | · | · | · | I | R | H | D |
| ZM | D | · | · | · | · | · | · | · | · | I | R | H | D |
| XC | D | · | · | · | · | · | · | · | · | I | R | H | D |
| HN2012 | D | · | · | · | · | · | · | · | · | I | R | H | D |
| HNX | D | · | · | · | · | · | · | · | · | I | R | H | D |
| HNB | D | · | · | · | · | · | · | · | · | I | R | H | D |
| TJ | D | · | · | · | · | · | · | · | · | · | R | H | D |
| ZJ01 | D | · | R | · | · | · | · | · | · | I | R | H | D |
| JS-2012 | D | · | · | · | · | · | · | · | · | I | R | H | D |
| LA | D | · | · | · | · | · | · | · | · | · | R | H | D |
| Fa | D | · | · | · | T | · | R | · | · | · | R | H | D |
| Ea | D | · | · | · | · | · | · | P | · | · | R | H | · |
| SC | D | · | · | · | · | · | · | P | · | · | R | H | · |
| Hercules | · | · | · | · | · | · | · | · | G | · | · | · | · |
| Kaplan | · | · | · | · | · | · | · | · | G | · | · | · | · |
| Kolchis | · | T | · | · | · | · | · | · | G | · | · | · | · |

Continue

| Strain | Amino acid point mutation positions (position of alignment) | | | | | | | | | | | | |
| --- | --- | --- | --- | --- | --- | --- | --- | --- | --- | --- | --- | --- | --- |
|  | 498 | 502 | 503 | 504 | 509 | 512 | 520 | 522 | 526 | 532 | 537 | 544 | 573 |
| Becker | —— | G | D | A | S | G | P | V | A | S | G | E | S |
| BP | · | · | V | I | A | S | · | A | P | · | · | · | N |
| GY | · | · | V | I | A | S | · | A | P | · | · | · | N |
| JY | · | · | V | I | A | S | · | A | P | · | · | · | N |
| LGX | · | · | V | I | A | S | · | A | P | · | · | · | N |
| M5 | · | · | V | I | A | S | · | A | P | · | · | · | N |
| MZ1 | · | · | V | I | A | S | · | A | P | · | · | · | N |
| MZ2 | · | · | V | I | A | S | · | A | P | · | · | · | N |
| NY | · | · | V | I | A | S | · | A | P | · | · | · | N |
| SMX | · | · | V | I | A | S | · | A | P | · | · | · | N |
| WY | · | · | V | I | A | S | · | A | P | · | · | · | N |
| WZ | · | · | V | I | A | S | · | A | P | · | · | · | N |
| YY | · | · | V | I | A | · | · | A | P | · | · | · | N |
| YZ | · | · | V | I | A | S | · | A | P | · | · | · | N |
| ZK | · | · | V | I | A | S | · | A | P | G | · | · | N |
| ZM | · | · | V | I | A | S | · | A | P | · | · | · | N |
| XC | · | · | V | I | A | S | · | A | P | · | · | · | N |
| HN2012 | · | · | V | I | A | S | · | A | P | · | · | · | N |
| HNX | · | · | V | I | A | S | · | A | P | · | · | · | N |
| HNB | · | · | V | I | A | S | · | A | P | · | · | · | N |
| TJ | · | · | V | I | A | · | · | A | P | · | · | · | N |
| ZJ01 | · | · | V | I | A | S | · | A | P | · | · | · | N |
| JS-2012 | · | · | V | I | A |  | · | A | P | · | · | · | N |
| LA | · | · | V | I | A | · | · | A | P | · | · | · | N |
| Fa | D | · | V | I | A | · | · | A | P | · | E | K | N |
| Ea | · | S | V | I | A | · | S | A | P | · | · | · | N |
| SC | · | S | V | I | A | · | S | A | P | · | · | · | N |
| Hercules | · | · | V | · | · | · | · | · | · | · | · | · | N |
| Kaplan | · | · | V | · | · | · | · | · | · | · | · | · | N |
| Kolchis | · | · | V | · | · | · | · | · | · | · | · | · | · |

Continue

| Strain | Amino acid point mutation positions (position of alignment) | | | | |
| --- | --- | --- | --- | --- | --- |
|  | 577 | 578 | 579 | 581 |  |
| Becker | N | A | R | A |  |
| BP | I | S | · | · |  |
| GY | M | S | · | · |  |
| JY | M | S | · | · |  |
| LGX | M | S | · | · |  |
| M5 | M | S | · | · |  |
| MZ1 | M | S | · | · |  |
| MZ2 | M | S | · | · |  |
| NY | M | S | · | · |  |
| SMX | M | S | · | · |  |
| WY | M | S | · | · |  |
| WZ | M | S | · | · |  |
| YY | M | S | · | · |  |
| YZ | M | S | · | · |  |
| ZK | M | S | · | · |  |
| ZM | M | S | · | · |  |
| XC | M | S | · | · |  |
| HN2012 | M | S | · | · |  |
| HNX | M | S | · | · |  |
| HNB | M | S | · | · |  |
| TJ | M | S | · | · |  |
| ZJ01 | H | V | P | R |  |
| JS-2012 | M | S | · | · |  |
| LA | M | S | · | · |  |
| Fa | · | · | · | · |  |
| Ea | M | S | · | · |  |
| SC | · | S | V | I |  |
| Hercules | · | · | V | · |  |
| Kaplan | · | · | V | · |  |
| Kolchis | · | · | V | · |  |
