# Supplementary material for: Isolation and Phylogenetic Analysis of Reemerging Pseudorabies Virus Within Pig Populations in Central China During 2012 to 2019
Source: Front Vet Sci. 2021 Nov 16;8:764982. doi: 10.3389/fvets.2021.764982 (PMC8635136; doi:10.3389/fvets.2021.764982)
Supplement: Supplementary file 2 [file Table_2.DOCX]

Supplementary Table 2 Amino acid (AA) mutations of gC protein of 16 PRV isolates from this study and 14 PRV reference strains compared with Bartha strain

| Strain | Amino acid point mutation positions (position of alignment) | | | | | | | | | | | | |
| --- | --- | --- | --- | --- | --- | --- | --- | --- | --- | --- | --- | --- | --- |
|  | 3 | 6 | 14 | 16 | 25 | 28 | 30 | 34 | 43 | 52 | 55 | 57 | 59 |
| Bartha | S | R | P | A | T | A | G | N | A | P | A | A | P |
| BP | **·** | **·** | **L** | T | S | **·** | **·** | T | **·** | S | E | V | G |
| GY | **·** | **·** | **L** | T | S | **·** | **·** | T | **·** | S | E | V | G |
| JY | **·** | **·** | **L** | T | S | **·** | **·** | **·** | **·** | S | E | V | G |
| LGX | **·** | **·** | **L** | T | S | **·** | **·** | **·** | **·** | S | E | V | G |
| M5 | **·** | **·** | **L** | T | S | **·** | **·** | **·** | **·** | S | E | V | G |
| MZ1 | **·** | **·** | **L** | T | S | **·** | **·** | **·** | **·** | S | E | V | G |
| MZ2 | **·** | **·** | **L** | T | S | **·** | **·** | **·** | **·** | S | E | V | G |
| NY | **·** | **·** | **L** | T | S | **·** | **·** | **·** | **·** | S | E | V | G |
| SMX | **·** | C | **L** | T | S | **·** | **·** | **·** | **·** | S | E | V | G |
| WY | **·** | **·** | **L** | T | S | **·** | **·** | **·** | **·** | **·** | E | V | G |
| WZ | **·** | **·** | **L** | T | S | **·** | **·** | T | **·** | S | E | V | G |
| YY | **·** | **·** | **L** | T | S | **·** | **·** | **·** | **·** | S | E | V | G |
| YZ | **·** | **·** | **L** | T | S | **·** | **·** | **·** | **·** | S | E | V | G |
| ZK | **·** | **·** | **L** | T | S | **·** | **·** | **·** | **·** | S | E | V | G |
| ZM | **·** | **·** | **L** | T | S | **·** | **·** | **·** | **·** | S | E | V | G |
| XC | P | **·** | **L** | T | S | **·** | **·** | **·** | **·** | S | E | V | G |
| HN2012 | **·** | **·** | **L** | T | S | **·** | **·** | **·** | **·** | S | E | V | G |
| HNX | **·** | **·** | **L** | T | S | **·** | **·** | **·** | **·** | S | E | V | G |
| HNB | **·** | **·** | **L** | T | S | **·** | **·** | **·** | **·** | S | E | V | G |
| TJ | **·** | **·** | **L** | T | S | **·** | **·** | **·** | **·** | S | E | V | G |
| ZJ01 | **·** | **·** | **L** | T | S | **·** | **·** | **·** | **·** | S | E | V | G |
| JS-2012 | **·** | **·** | **L** | T | S | **·** | **·** | **·** | **·** | S | E | V | G |
| LA | **·** | **·** | **L** | T | **·** | **·** | **·** | **·** | **·** | S | — | — | — |
| Fa | **·** | **·** | **L** | T | **·** | V | **·** | **·** | **·** | S | E | V | G |
| Ea | **·** | **·** | **L** | T | S | **·** | **·** | T | **·** | S | E | V | G |
| SC | **·** | **·** | **·** | **·** | **·** | **·** | **·** | **·** | **·** | S | **·** | **·** | **·** |
| Becker | **·** | **·** | **L** | **·** | **·** | **·** | D | **·** | E | S | **·** | **·** | **·** |
| Hercules | **·** | **·** | **L** | **·** | **·** | **·** | **·** | **·** | E | S | **·** | **·** | **·** |
| Kaplan | **·** | **·** | **L** | **·** | **·** | **·** | **·** | **·** | E | S | **·** | **·** | **·** |
| Kolchis | **·** | **·** | **L** | **·** | **·** | **·** | **·** | **·** | E | S | **·** | **·** | **·** |

Continue

| Strain | Amino acid point mutation positions (position of alignment) | | | | | | | | | | | | |
| --- | --- | --- | --- | --- | --- | --- | --- | --- | --- | --- | --- | --- | --- |
|  | 60 | 61 | 63 | 64 | 65 | 66 | 67 | 68 | 69 | 70 | 72 | 75 | 76 |
| Bartha | E | A | —— | —— | —— | —— | —— | —— | —— | A | S | R | A |
| BP | T | T | A | A | A | S | T | P | A | · | L | · | V |
| GY | T | T | A | A | A | S | T | P | A | · | L | · | V |
| JY | T | T | A | A | A | S | T | P | A | · | · | · | V |
| LGX | T | T | A | A | A | S | T | P | A | · | · | · | V |
| M5 | T | T | A | A | A | S | T | P | A | · | · | · | V |
| MZ1 | T | T | A | A | A | S | T | P | A | · | · | · | V |
| MZ2 | T | T | A | A | A | S | T | P | A | · | · | · | V |
| NY | T | T | A | A | A | S | T | P | A | · | · | · | V |
| SMX | T | T | A | A | A | S | T | P | A | · | · | · | V |
| WY | T | T | A | A | A | S | T | P | A | T | · | · | V |
| WZ | T | T | A | A | A | S | T | P | A | · | · | · | V |
| YY | T | T | A | A | A | S | T | P | A | · | · | · | V |
| YZ | T | T | A | A | A | S | T | P | A | · | · | · | V |
| ZK | T | T | A | A | A | S | T | P | A | · | · | · | V |
| ZM | T | T | A | A | A | S | T | P | A | · | · | · | V |
| XC | T | T | A | A | A | S | T | P | A | · | · | · | V |
| HN2012 | T | T | A | A | A | S | T | P | A | · | · | · | V |
| HNX | T | T | A | A | A | S | T | P | A | · | · | · | V |
| HNB | T | T | A | A | A | S | T | P | A | · | · | · | V |
| TJ | T | T | A | A | A | S | T | P | A | · | · | · | V |
| ZJ01 | T | T | A | A | A | S | T | P | A | · | · | · | V |
| JS-2012 | T | T | A | A | A | S | T | P | A | · | · | · | V |
| LA | —— | —— | A | A | A | S | T | P | A | · | · | · | V |
| Fa | T | T | A | A | A | S | T | P | A | · | · | P | V |
| Ea | T | T | A | A | A | S | T | P | A | · | · | · | V |
| SC | · | · | A | A | A | S | T | P | A | · | · | · | · |
| Becker | · | · | —— | —— | —— | —— | —— | —— | —— | · | · | P | V |
| Hercules | · | · | —— | —— | —— | —— | —— | —— | —— | · | · | · | · |
| Kaplan | · | · | —— | —— | —— | —— | —— | —— | —— | · | · | · | · |
| Kolchis | · | · | —— | —— | —— | —— | —— | —— | —— | · | · | · | · |

Continue

| Strain | Amino acid point mutation positions (position of alignment) | | | | | | | | | | | | |
| --- | --- | --- | --- | --- | --- | --- | --- | --- | --- | --- | --- | --- | --- |
|  | 87 | 90 | 99 | 102 | 103 | 106 | 107 | 130 | 139 | 142 | 163 | 186 | 187 |
| Bartha | P | N | K | A | H | K | R | F | V | Y | S | T | P |
| BP | Q | G | E | S | · | · | · | V | · | C | P | A | S |
| GY | Q | G | E | S | · | · | · | V | · | C | P | A | S |
| JY | Q | G | · | S | · | · | · | V | · | C | P | A | S |
| LGX | Q | G | · | S | · | · | · | V | · | C | P | A | S |
| M5 | Q | G | · | S | · | · | · | V | · | C | P | A | S |
| MZ1 | Q | G | · | S | · | · | · | V | · | C | P | A | S |
| MZ2 | Q | G | · | S | · | · | H | V | · | C | P | A | S |
| NY | Q | G | · | S | · | · | · | V | · | C | P | A | S |
| SMX | Q | G | · | S | · | · | · | V | · | C | P | A | S |
| WY | Q | G | · | S | · | · | · | V | · | C | P | A | S |
| WZ | Q | G | E | S | · | · | · | V | I | C | P | A | S |
| YY | Q | G | · | S | · | · | · | V | · | C | P | A | S |
| YZ | Q | G | · | S | · | · | · | V | · | C | P | A | S |
| ZK | Q | G | · | S | · | · | · | V | · | C | P | A | S |
| ZM | Q | G | · | S | · | · | · | V | · | C | P | A | S |
| XC | Q | G | · | S | · | · | · | V | · | C | P | A | S |
| HN2012 | Q | G | · | S | · | · | · | V | · | C | P | A | S |
| HNX | Q | G | · | S | · | · | · | V | · | C | P | A | S |
| HNB | Q | G | · | S | · | · | · | V | · | C | P | A | S |
| TJ | Q | G | · | S | · | · | · | V | · | C | P | A | S |
| ZJ01 | Q | G | · | S | · | · | · | V | · | C | P | A | S |
| JS-2012 | Q | G | · | S | · | · | · | V | · | C | P | A | S |
| LA | Q | G | · | S | · | · | · | V | · | C | P | A | S |
| Fa | Q | G | R | S | R | · | · | V | · | · | P | A | S |
| Ea | Q | G | E | S | · | · | · | V | · | C | P | A | S |
| SC | · | · | · | · | · | E | · | · | · | · | · | · | · |
| Becker | · | · | · | · | · | · | · | · | · | · | P | A | · |
| Hercules | · | · | · | · | · | · | · | · | · | · | P | A | · |
| Kaplan | · | · | · | · | · | E | · | · | · | · | P | · | · |
| Kolchis | · | · | · | · | · | · | · | · | · | · | P | A | · |

Continue

| Strain | Amino acid point mutation positions (position of alignment) | | | | | | | | | | | | |
| --- | --- | --- | --- | --- | --- | --- | --- | --- | --- | --- | --- | --- | --- |
|  | 188 | 189 | 190 | 191 | 194 | 203 | 204 | 240 | 243 | 280 | 300 | 303 | 324 |
| Bartha | V | V | E | D | G | G | E | L | S | F | L | R | D |
| BP | A | · | V | V | · | · | · | V | H | · | · | · | · |
| GY | A | · | V | V | · | · | · | V | H | L |  |  | · |
| JY | A | · | V | V | E | · | · | V | H | · | · | · | · |
| LGX | A | · | V | V | E | · | · | V | H | L | · | · | · |
| M5 | A | · | V | V | E | · | · | V | H | · | · | · | · |
| MZ1 | A | · | V | V | E | R | · | V | H | L | · | · | · |
| MZ2 | A | · | V | V | E | · | · | V | H | L | · | · | · |
| NY | A | · | V | V | E | · | · | V | H | L | · | · | · |
| SMX | A | · | V | V | E | · | · | V | H | · | F | · | · |
| WY | A | · | V | V | E | · | · | V | H | · | · | · | · |
| WZ | A | · | V | V | · | · | · | V | H | · | · | · | · |
| YY | A | · | V | V | E | · | · | V | H | L | · | · | · |
| YZ | A | · | V | V | E | · | · | V | H | · | · | · | · |
| ZK | A | · | V | V | E | · | · | V | H | L | · | · | · |
| ZM | A | · | V | V | E | · | · | V | H | L | · | · | · |
| XC | A | · | V | V | E | · | · | V | H | · | · | · | · |
| HN2012 | A | · | V | V | E | · | · | V | H | · | · | · | · |
| HNX | A | · | V | V | E | · | · | V | H | · | · | · | · |
| HNB | A | · | V | V | E | · | · | V | H | · | · | · | · |
| TJ | A | · | V | V | E | · | · | V | H | · | · | · | · |
| ZJ01 | A | · | V | V | E | · | · | V | H | · | · | · | · |
| JS-2012 | A | · | V | V | E | · | · | V | H | · | · | H | · |
| LA | A | · | V | V | E | · | · | V | H | · | · | · | · |
| Fa | A | · | V | V | E | · | K | V | H | · | · | · | · |
| Ea | A | · | V | V | · | · | · | V | H | · | · | · | · |
| SC | · | · | · | · | · | · | · | · | · | · | · | · | · |
| Becker | · | · | V | E | E | · | · | · | · | · | · | · | A |
| Hercules | A | L | D | · | · | · | · | · | · | · | · | · | · |
| Kaplan | A | · | · | · | · | · | · | · | · | · | · | · | · |
| Kolchis | A | L | D | · | · | · | · | · | · | · | · | · | · |

Continue

| Strain | Amino acid point mutation positions (position of alignment) | | | | | | | | | | | | |
| --- | --- | --- | --- | --- | --- | --- | --- | --- | --- | --- | --- | --- | --- |
|  | 359 | 365 | 389 | 399 | 403 | 431 | 437 | 449 | 457 | 461 | 462 | 467 | 468 |
| Bartha | p | R | H | A | H | L | V | A | S | V | V | G | I |
| BP | · | · | · | · | · | M | I | T | T | T | · | A | · |
| GY | · | · | · | · | · | M | I | T | T | T | · | A | · |
| JY | · | · | · | · | · | M | I | T | T | T | · | A | · |
| LGX | · | · | · | · | · | M | I | T | T | T | · | A | · |
| M5 | · | · | · | · | · | M | I | T | T | T | · | A | · |
| MZ1 | · | · | Y | · | · | M | I | T | T | T | · | A | · |
| MZ2 | · | · | · | · | · | M | I | T | T | T | · | A | · |
| NY | · | · | · | · | · | M | I | T | T | T | · | A | · |
| SMX | · | · | · | V | · | M | I | T | T | T | · | A | · |
| WY | S | · | · | · | · | M | I | T | T | T | · | A | · |
| WZ | · | · | · | · | P | M | I | T | T | T | · | A | · |
| YY | · | · | · | · | · | M | I | T | T | T | · | A | · |
| YZ | · | · | · | · | · | M | I | T | T | T | A | A | · |
| ZK | · | L | · | · | · | M | I | T | T | T | · | A | · |
| ZM | · | · | · | · | · | M | I | T | T | T | · | A | · |
| XC | · | · | · | · | · | M | I | T | T | T | · | A | T |
| HN2012 | · | · | · | · | · | M | I | T | T | T | · | A | · |
| HNX | · | · | · | · | · | M | I | T | T | T | · | A | · |
| HNB | · | · | · | · | · | M | I | T | T | T | · | A | · |
| TJ | · | · | · | · | · | M | I | T | T | T | · | A | · |
| ZJ01 | · | · | · | · | · | M | I | T | T | T | · | A | · |
| JS-2012 | · | · | · | · | · | M | I | T | T | T | · | A | · |
| LA | · | · | · | · | · | M | · | T | T | T | · | A | · |
| Fa | · | · | · | · | · | M | · | T | T | T | · | A | · |
| Ea | · | · | · | · | · | M | I | T | T | T | · | A | · |
| SC | · | · | · | · | · | M | I | T | T | T | · | A | · |
| Becker | · | · | · | · | · | · | · | · | · | · | · | · | · |
| Hercules | · | · | · | · | · | · | · | · | · | · | · | · | · |
| Kaplan | · | · | · | · | · | · | · | · | · | · | · | · | · |
| Kolchis | · | · | · | · | · | · | · | · | · | · | · | · | · |

Continue

| Strain | Amino acid point mutation positions (position of alignment) | | | | |
| --- | --- | --- | --- | --- | --- |
|  | 484 | 485 | 486 | 487 |  |
| Bartha | R | A | G | P |  |
| BP | · | S | A | L |  |
| GY | · | S | A | L |  |
| JY | · | S | A | L |  |
| LGX | · | S | A | L |  |
| M5 | · | S | A | L |  |
| MZ1 | · | S | A | L |  |
| MZ2 | · | S | A | L |  |
| NY | · | S | A | L |  |
| SMX | · | S | A | L |  |
| WY | · | S | A | L |  |
| WZ | · | S | A | L |  |
| YY | · | S | A | L |  |
| YZ | · | S | A | L |  |
| ZK | · | S | A | L |  |
| ZM | · | S | A | L |  |
| XC | · | S | A | L |  |
| HN2012 | · | S | A | L |  |
| HNX | · | S | A | L |  |
| HNB | · | S | A | L |  |
| TJ | · | S | A | L |  |
| ZJ01 | · | S | A | L |  |
| JS-2012 | · | S | A | L |  |
| LA | · | P | V | L |  |
| Fa | · | P | V | L |  |
| Ea | · | S | A | L |  |
| SC | · | S | A | L |  |
| Becker | Q | S | A | L |  |
| Hercules | · | · | · | · |  |
| Kaplan | · | · | · | · |  |
| Kolchis | · | · | · | · |  |
