# Supplementary material for: Isolation and Phylogenetic Analysis of Reemerging Pseudorabies Virus Within Pig Populations in Central China During 2012 to 2019
Source: Front Vet Sci. 2021 Nov 16;8:764982. doi: 10.3389/fvets.2021.764982 (PMC8635136; doi:10.3389/fvets.2021.764982)
Supplement: Supplementary file 3 [file Table_3.DOCX]

Supplementary Table 3 Amino acid (AA) mutations of gB protein of 16 PRV isolates from this study and 13 PRV reference strains compared with Bartha strain

| Strain | Amino acid point mutation positions ( position of alignment) | | | | | | | | | | |
| --- | --- | --- | --- | --- | --- | --- | --- | --- | --- | --- | --- |
|  | 7 | 43 | 50 | 52 | 53 | 55 | 57 | 61 | 70 | 72 | 73 |
| Bartha | L | L | L | A | A | P | G | V | T | V | P |
| BP | · | · | · | · | T | T | · | · | A | G | T |
| GY | · | · | P | · | T | T | · | · | A | G | T |
| JY | · | R | · | V | T | T | S | · | A | G | T |
| LGX | · | · | · | · | T | T | · | · | A | G | T |
| M5 | P | · | · | · | T | T | · | · | A | G | T |
| MZ1 | · | · | · | · | T | T | · | · | A | G | T |
| MZ2 | · | · | · | · | T | T | · | M | A | G | T |
| NY | · | · | · | · | T | T | · | · | A | G | T |
| SMX | · | · | · | · | T | T | · | · | A | G | T |
| WY | · | · | · | · | T | T | · | · | A | G | T |
| WZ | · | · | · | · | T | T | · | · | A | G | T |
| YY | · | · | · | · | T | T | · | · | A | G | T |
| YZ | · | · | · | · | T | T | · | · | A | G | T |
| ZK | · | · | · | · | T | T | · | · | A | G | T |
| ZM | · | · | · | · | T | T | · | · | A | G | T |
| XC | · | · | · | · | T | T | · | · | A | G | T |
| HN2012 | · | · | · | · | T | T | · | · | A | G | T |
| HNX | · | · | · | · | T | T | · | · | A | G | T |
| HNB | · | · | · | · | T | T | · | · | A | G | T |
| TJ | · | · | · | · | T | T | · | · | A | G | T |
| ZJ01 | · | · | · | · | T | T | · | · | A | G | T |
| JS-2012 | · | · | · | · | T | T | · | · | A | G | T |
| LA | · | · | · | · | T | T | · | · | A | G | T |
| Ea | · | · | · | · | T | T | · | · | A | G | T |
| SC | · | · | · | · | T | T | · | · | A | G | T |
| Becker | · | · | · | · | · | · | · | · | · | G | T |
| Hercules | · | · | · | · | · | · | · | · | · | · | · |
| Kaplan | · | · | · | · | · | · | · | · | · | · | · |
| Kolchis | · | · | · | · | · | · | · | · | · | · | · |

Continue

| Strain | Amino acid point mutation positions ( position of alignment) | | | | | | | | | | |
| --- | --- | --- | --- | --- | --- | --- | --- | --- | --- | --- | --- |
|  | 75 | 76 | 77 | 78 | 81 | 81 | 83 | 85 | 87 | 93 | 94 |
| Bartha | S | P | G | L | N | D | V | A | A | E | —— |
| BP | —— | —— | —— | A | D | G | F | T | E | D | G |
| GY | —— | —— | —— | A | D | G | F | · | E | D | G |
| JY | —— | —— | —— | A | D | G | F | · | E | D | G |
| LGX | —— | —— | —— | A | D | G | F | · | E | D | G |
| M5 | —— | —— | —— | A | D | G | F | · | E | D | G |
| MZ1 | —— | —— | —— | A | D | G | F | · | E | D | G |
| MZ2 | —— | —— | —— | A | D | G | F | · | E | D | G |
| NY | —— | —— | —— | A | D | G | F | · | E | D | G |
| SMX | —— | —— | —— | A | D | G | F | T | E | D | G |
| WY | —— | —— | —— | A | D | G | F | T | E | D | G |
| WZ | —— | —— | —— | A | D | G | F | T | E | D | G |
| YY | —— | —— | —— | A | D | G | F | T | E | D | G |
| YZ | —— | —— | —— | A | G | G | F | T | E | D | G |
| ZK | —— | —— | —— | A | D | G | F | · | E | D | G |
| ZM | —— | —— | —— | A | D | G | F | · | E | D | G |
| XC | —— | —— | —— | A | D | G | F | · | E | D | G |
| HN2012 | —— | —— | —— | A | D | G | F | · | E | D | G |
| HNX | —— | —— | —— | A | D | G | F | · | E | D | G |
| HNB | —— | —— | —— | A | D | G | F | · | E | D | G |
| TJ | —— | —— | —— | A | D | G | F | · | E | D | G |
| ZJ01 | —— | —— | —— | A | D | G | F | · | E | D | G |
| JS-2012 | —— | —— | —— | A | D | G | F | · | E | D | G |
| LA | —— | —— | —— | A | D | G | F | · | E | D | G |
| Ea | —— | —— | —— | A | D | G | F | T | E | D | G |
| SC | —— | —— | —— | A | D | G | F | T | E | D | G |
| Becker | —— | —— | —— | A | · | · | · | · | · | · | · |
| Hercules | · | · | · | · | · | · | · | · | · | · | · |
| Kaplan | · | · | · | · | · | · | · | · | · | · | · |
| Kolchis | · | · | · | · | · | · | · | · | · | · | · |

Continue

| Strain | Amino acid point mutation positions ( position of alignment) | | | | | | | | | | |
| --- | --- | --- | --- | --- | --- | --- | --- | --- | --- | --- | --- |
|  | 96 | 97 | 102 | 133 | 138 | 145 | 149 | 153 | 158 | 187 | 190 |
| Bartha | F | T | E | P | V | Q | E | G | G | V | G |
| BP | V | S | D | · | · | · | · | · | · | · | · |
| GY | V | S | D | · | · | P | · | · | · | · | · |
| JY | V | S | D | · | · | · | · | · | · | · | · |
| LGX | V | S | D | S | · | · | · | · | · | · | · |
| M5 | V | S | D | · | · | · | · | · | · | · | · |
| MZ1 | V | S | D | · | · | · | · | · | · | · | · |
| MZ2 | V | S | D | · | · | · | · | E | G | · | · |
| NY | V | S | D | · | · | T | · | · | · | · | · |
| SMX | V | S | D | · | · | · | · | · | · | A | · |
| WY | V | S | D | · | · | · | · | · | · | · | · |
| WZ | V | S | D | · | · | · | · | · | · | · | E |
| YY | V | S | D | · | A | · | · | · | · | · | · |
| YZ | V | S | D | · | · | · | · | · | · | · | · |
| ZK | V | S | D | · | · | · | · | · | · | · | · |
| ZM | V | S | D | · | · | · | · | · | · | · | · |
| XC | V | S | D | · | · | · | · | · | · | · | · |
| HN2012 | V | S | D | · | · | · | · | · | · | · | · |
| HNX | V | S | D | · | · | · | · | · | · | · | · |
| HNB | V | S | D | · | · | · | · | · | · | · | · |
| TJ | V | S | D | · | · | · | · | · | · | · | · |
| ZJ01 | V | S | D | · | · | · | Q | · | · | · | · |
| JS-2012 | V | S | D | · | · | · | · | · | · | · | · |
| LA | V | S | · | · | · | · | · | · | · | · | · |
| Ea | V | S | D | · | · | · | · | · | · | · | · |
| SC | V | S | D | · | · | · | · | · | · | · | · |
| Becker | · | S | · | · | · | · | · | · | · | · | · |
| Hercules | · | · | · | · | · | · | · | · | · | · | · |
| Kaplan | · | · | · | · | · | · | · | · | · | · | · |
| Kolchis | · | · | · | · | · | · | · | · | · | · | · |

Continue

| Strain | Amino acid point mutation positions ( position of alignment) | | | | | | | | | | |
| --- | --- | --- | --- | --- | --- | --- | --- | --- | --- | --- | --- |
|  | 209 | 262 | 268 | 280 | 286 | 287 | 290 | 307 | 338 | 396 | 405 |
| Bartha | Q | H | H | G | I | V | V | D | H | D | A |
| BP | · | · | Y | · | · | · | · | · | · | · | · |
| GY | · | · | Y | S | · | · | A | · | · | · | · |
| JY | · | · | Y | · | · | · | · | G | R | · | · |
| LGX | · | · | Y | · | · | A | · | · | · | · | · |
| M5 | · | · | Y | · | · | · | · | · | · | · | · |
| MZ1 | · | · | Y | · | · | · | · | · | · | · | · |
| MZ2 | · | R | Y | · | · | · | · | · | · | · | · |
| NY | · | · | Y | · | · | · | · | · | · | · | · |
| SMX | · | · | Y | · | · | · | · | · | · | · | · |
| WY | R | · | Y | · | · | · | · | · | · | · | · |
| WZ | · | · | Y | · | · | · | · | · | · | · | T |
| YY | · | · | Y | · | V | · | · | · | · | · | · |
| YZ | · | · | Y | · | · | · | · | · | · | · | · |
| ZK | · | · | Y | · | · | · | · | · | · | · | · |
| ZM | · | · | Y | · | · | · | · | · | · | · | · |
| XC | · | · | Y | · | · | · | · | · | · | · | · |
| HN2012 | · | · | Y | · | · | · | · | · | · | · | · |
| HNX | · | · | Y | · | · | · | · | · | · | · | · |
| HNB | · | · | Y | · | · | · | · | · | · | · | · |
| TJ | · | · | Y | · | · | · | · | · | · | · | · |
| ZJ01 | · | · | Y | · | · | · | · | · | · | · | · |
| JS-2012 | · | · | Y | · | · | · | · | · | · | · | · |
| LA | · | · | Y | · | · | · | · | Y | · | · | · |
| Ea | · | · | Y | · | · | · | · | · | · | G | · |
| SC | · | · | Y | · | · | · | · | · | · | · | · |
| Becker | · | · | Y | · | · | · | · | · | · | · | · |
| Hercules | · | · | Y | · | · | · | · | · | · | · | · |
| Kaplan | · | · | Y | · | · | · | · | · | · | · | · |
| Kolchis | · | · | Y | · | · | · | · | · | · | · | · |

Continue

| Strain | Amino acid point mutation positions ( position of alignment) | | | | | | | | | | |
| --- | --- | --- | --- | --- | --- | --- | --- | --- | --- | --- | --- |
|  | 417 | 436 | 441 | 446 | 454 | 459 | 497 | 502 | 507 | 508 | 510 |
| Bartha | L | I | Q | N | R | L | S | R | A | A | Q |
| BP | · | · | R | · | · | · | · | · | S | P | P |
| GY | P | · | R | · | K | · | · | C | S | P | P |
| JY | · | · | R | · | K | · | · | · | S | P | P |
| LGX | · | · | R | · | K | · | · | · | S | P | P |
| M5 | · | · | R | · | K | · | · | · | S | P | P |
| MZ1 | · | · | R | · | K | · | · | · | S | P | P |
| MZ2 | · | · | R | · | K | · | · | · | S | P | P |
| NY | · | · | R | · | K | · | · | · | S | P | P |
| SMX | · | · | R | · | · | · | · | · | S | P | P |
| WY | · | · | R | · | · | · | · | · | S | P | P |
| WZ | · | · | R | · | · | P | · | · | S | P | P |
| YY | · | · | R | · | · | · | · | · | S | P | P |
| YZ | · | · | R | · | · | · | · | · | S | P | P |
| ZK | · | · | R | · | K | · | · | · | S | P | P |
| ZM | · | · | R | · | K | · | · | · | S | P | P |
| XC | · | · | R | · | K | · | · | · | S | P | P |
| HN2012 | · | · | R | · | K | · | · | · | S | P | P |
| HNX | · | · | R | · | K | · | · | · | S | P | P |
| HNB | · | · | R | · | K | · | · | · | S | P | P |
| TJ | · | · | R | · | K | · | · | · | S | P | P |
| ZJ01 | · | · | R | · | K | · | · | · | S | P | P |
| JS-2012 | · | · | R | · | K | · | · | · | S | P | P |
| LA | · | V | R | · | · | · | · | · | S | · | · |
| Ea | · | · | R | · | · | · | · | · | S | P | P |
| SC | · | · | R | · | · | · | · | · | S | P | P |
| Becker | · | · | R | S | · | · | A | · | S | P | P |
| Hercules | · | · | R | · | · | · | · | · | · | · | · |
| Kaplan | · | · | R | · | · | · | · | · | S | P | P |
| Kolchis | · | · | R | · | · | · | · | · | · | · | · |

Continue

| Strain | Amino acid point mutation positions ( position of alignment) | | | | | | | | | | |
| --- | --- | --- | --- | --- | --- | --- | --- | --- | --- | --- | --- |
|  | 511 | 523 | 526 | 553 | 554 | 557 | 563 | 571 | 605 | 614 | 627 |
| Bartha | A | G | R | G | R | A | Q | S | V | Q | Y |
| BP | · | · | · | S | · | · | H | G | · | · | · |
| GY | · | · | · | S | · | · | · | G | · | · | · |
| JY | · | · | · | S | · | · | · | G | · | · | · |
| LGX | V | · | H | S | · | · | · | G | · | · | · |
| M5 | · | · | · | S | · | · | · | G | · | · | · |
| MZ1 | · | · | · | S | · | · | · | G | · | · | · |
| MZ2 | · | · | · | S | · | · | · | G | · | · | · |
| NY | · | · | · | S | · | · | · | G | · | · | · |
| SMX | · | · | · | S | · | · | H | G | · | · | · |
| WY | · | · | · | S | · | · | H | G | · | · | · |
| WZ | · | · | · | S | · | · | H | G | · | · | · |
| YY | · | W | · | S | · | · | H | G | M | · | · |
| YZ | · | · | · | S | · | · | H | G | · | · | · |
| ZK | · | · | · | S | C | · | · | G | · | R | · |
| ZM | · | · | · | S | C | · | · | G | · | R | · |
| XC | · | · | · | S | · | · | · | G | · | · | · |
| HN2012 | · | · | · | S | · | · | · | G | · | · | · |
| HNX | · | · | · | S | · | · | · | G | · | · | · |
| HNB | · | · | · | S | · | · | · | G | · | · | · |
| TJ | · | · | · | S | · | · | · | G | · | · | · |
| ZJ01 | · | · | · | S | · | · | · | G | · | · | · |
| JS-2012 | · | · | · | S | · | · | · | G | · | · | · |
| LA | · | · | · | S | · | · | · | G | · | · | · |
| Ea | · | · | · | S | · | · | H | G | · | · | · |
| SC | · | · | · | S | · | · | H | G | · | · | · |
| Becker | · | · | · | · | · | · | · | · | · | · | · |
| Hercules | · | · | · | · | · | · | · | · | · | · | · |
| Kaplan | · | · | · | · | · | T | · | · | · | · | · |
| Kolchis | · | · | · | · | · | · | · | · | · | · | · |

Continue

| Strain | Amino acid point mutation positions ( position of alignment) | | | | | | | | | | |
| --- | --- | --- | --- | --- | --- | --- | --- | --- | --- | --- | --- |
|  | 636 | 674 | 683 | 684 | 696 | 710 | 718 | 738 | 740 | 750 | 765 |
| Bartha | H | S | S | Y | T | F | R | L | A | V | F |
| BP | L | G | · | · | · | · | P | · | T | · | · |
| GY | · | G | · | · | · | · | · | · | · | · | · |
| JY | · | G | · | C | · | · | · | · | · | A | · |
| LGX | · | G | · | · | · | · | · | · | · | · | · |
| M5 | · | G | · | · | · | · | · | · | · | · | · |
| MZ1 | · | G | · | · | M | · | · | · | · | · | · |
| MZ2 | · | G | · | · | · | · | · | · | · | · | · |
| NY | · | G | · | · | · | · | · | · | · | · | · |
| SMX | · | G | · | · | · | · | · | · | T | · | · |
| WY | · | G | · | · | · | · | · | · | T | · | · |
| WZ | · | G | · | · | · | · | · | · | T | · | · |
| YY | · | G | · | · | · | · | · | · | T | · | L |
| YZ | · | G | · | · | · | · | · | · | T | · | · |
| ZK | · | G | · | · | · | · | · | · | · | · | · |
| ZM | · | G | · | · | · | · | · | · | · | · | · |
| XC | · | G | · | · | · | · | · | · | · | · | · |
| HN2012 | · | G | · | · | · | · | · | · | · | · | · |
| HNX | · | G | · | · | · | · | · | · | · | · | · |
| HNB | · | G | · | · | · | · | · | · | · | · | · |
| TJ | · | G | · | · | · | · | · | · | · | · | · |
| ZJ01 | · | G | · | · | · | · | · | · | · | · | · |
| JS-2012 | · | G | · | · | · | · | · | · | · | · | · |
| LA | · | G | · | · | · | · | · | · | · | · | · |
| Ea | · | G | · | · | · | · | · | P | T | · | · |
| SC | · | G | · | · | · | · | · | · | T | · | · |
| Becker | · | · | N | · | · | L | · | · | · | · | · |
| Hercules | · | · | · | · | · | · | · | · | · | · | · |
| Kaplan | · | · | · | · | · | · | · | · | · | · | · |
| Kolchis | · | · | · | · | · | · | · | · | · | · | · |

Continue

| Strain | Amino acid point mutation positions ( position of alignment) | | | | | | | | | | |
| --- | --- | --- | --- | --- | --- | --- | --- | --- | --- | --- | --- |
|  | 794 | 812 | 830 | 838 | 843 | 846 | 850 | 852 | 855 | 885 | 898 |
| Bartha | M | L | R | P | T | E | D | G | D | K | A |
| BP | T | · | · | · | A | · | E | D | · | · | V |
| GY | · | · | · | · | A | · | E | D | · | · | · |
| JY | · | · | · | H | A | · | E | D | · | · | · |
| LGX | · | · | · | · | A | V | E | D | · | · | · |
| M5 | · | · | · | · | A | · | E | D | · | · | · |
| MZ1 | · | · | H | · | A | · | E | D | · | · | · |
| MZ2 | · | · | H | · | A | · | E | D | · | · | · |
| NY | · | · | · | · | A | · | E | D | · | · | · |
| SMX | · | · | · | · | A | · | E | D | · | Q | V |
| WY | · | · | · | · | A | · | E | D | · | · | V |
| WZ | · | · | · | · | A | · | E | D | G | · | V |
| YY | · | P | · | · | A | · | E | D | · | · | V |
| YZ | · | · | · | · | A | · | E | D | · | · | V |
| ZK | · | · | · | · | A | · | E | D | · | · | · |
| ZM | · | · | · | · | A | · | E | D | · | · | · |
| XC | · | · | · | · | A | · | E | D | · | · | · |
| HN2012 | · | · | · | · | A | · | E | D | · | · | · |
| HNX | · | · | · | · | A | · | E | D | · | · | · |
| HNB | · | · | · | · | A | · | E | D | · | · | · |
| TJ | · | · | · | · | A | · | E | D | · | · | · |
| ZJ01 | · | · | · | · | A | · | E | D | · | · | · |
| JS-2012 | · | · | · | · | A | · | E | D | · | · | · |
| LA | · | · | · | · | A | · | E | · | · | · | · |
| Ea | · | · | · | · | A | · | E | D | · | · | V |
| SC | · | · | · | · | A | · | E | D | · | · | V |
| Becker | · | · | · | · | · | · | · | · | · | · | · |
| Hercules | · | · | · | · | · | · | · | · | · | · | · |
| Kaplan | · | · | · | · | · | · | · | · | · | · | · |
| Kolchis | · | · | · | · | · | · | · | · | · | · | · |

Continue

| Strain | Amino acid point mutation positions ( position of alignment) | | | | |
| --- | --- | --- | --- | --- | --- |
|  | 899 | 904 | 911 | 917 |  |
| Bartha | M | R | S | L |  |
| BP | **·** | **·** | N | P |  |
| GY | **·** | **·** | N | P |  |
| JY | **·** | **·** | N | P |  |
| LGX | **·** | W | N | P |  |
| M5 | **·** | **·** | N | P |  |
| MZ1 | **·** | **·** | N | P |  |
| MZ2 | **·** | **·** | N | P |  |
| NY | **·** | **·** | N | P |  |
| SMX | **·** | **·** | N | P |  |
| WY | **·** | **·** | N | P |  |
| WZ | **·** | **·** | N | S |  |
| YY | T | **·** | N | P |  |
| YZ | **·** | **·** | N | P |  |
| ZK | **·** | **·** | N | P |  |
| ZM | **·** | **·** | N | P |  |
| XC | **·** | **·** | N | P |  |
| HN2012 | **·** | **·** | N | P |  |
| HNX | **·** | **·** | N | P |  |
| HNB | **·** | **·** | N | P |  |
| TJ | **·** | **·** | N | P |  |
| ZJ01 | **·** | **·** | N | P |  |
| JS-2012 | **·** | **·** | N | P |  |
| LA | **·** | **·** | N | P |  |
| Ea | **·** | **·** | N | P |  |
| SC | **·** | **·** | N | P |  |
| Becker | **·** | **·** | **·** | **·** |  |
| Hercules | **·** | **·** | **·** | **·** |  |
| Kaplan | **·** | **·** | **·** | **·** |  |
| Kolchis | **·** | **·** | **·** | **·** |  |
