# Supplementary material for: Isolation and Phylogenetic Analysis of Reemerging Pseudorabies Virus Within Pig Populations in Central China During 2012 to 2019
Source: Front Vet Sci. 2021 Nov 16;8:764982. doi: 10.3389/fvets.2021.764982 (PMC8635136; doi:10.3389/fvets.2021.764982)
Supplement: Supplementary file 4 [file Table_4.DOCX]

Supplementary Table 4 Amino acid (AA) mutations of gD protein of 13 PRV isolates from this study and 14 PRV reference strains compared with Bartha strain

| Strain | Amino acid point mutation positions (position of alignment) | | | | | | | | | | |
| --- | --- | --- | --- | --- | --- | --- | --- | --- | --- | --- | --- |
|  | 12 | 69 | 82 | 117 | 122 | 143 | 169 | 170 | 207 | 209 | 212 |
| Bartha | A | A | S | C | I | F | S | V | N | E | R |
| BP | · | V | N | **·** | **·** | **·** | **·** | **·** | S | D | K |
| JY | · | V | N | **·** | **·** | **·** | **·** | **·** | S | D | K |
| LGX | · | V | N | **·** | **·** | **·** | **·** | **·** | S | D | K |
| MZ1 | · | V | N | **·** | **·** | **·** | **·** | **·** | S | D | K |
| MZ2 | · | V | N | **·** | **·** | **·** | **·** | **·** | S | D | K |
| NY | · | V | N | **·** | **·** | **·** | **·** | **·** | S | D | K |
| SMX | · | V | N | S | **·** | **·** | **·** | **·** | S | D | K |
| WY | · | V | N | **·** | **·** | **·** | **·** | **·** | S | D | K |
| WZ | · | V | N | **·** | **·** | **·** | **·** | **·** | S | D | K |
| YY | · | V | N | **·** | **·** | **·** | **·** | **·** | S | D | K |
| YZ | · | V | N | **·** | **·** | **·** | **·** | **·** | S | D | · |
| ZK | · | V | N | **·** | **·** | L | **·** | F | S | D | K |
| ZM | · | V | N | **·** | **·** | **·** | **·** | **·** | S | D | K |
| HN2012 | · | V | N | **·** | **·** | **·** | **·** | **·** | S | D | K |
| HNX | · | V | N | **·** | **·** | **·** | **·** | **·** | S | D | K |
| HNB | · | V | N | **·** | **·** | **·** | **·** | **·** | S | D | K |
| TJ | · | V | N | **·** | **·** | **·** | **·** | **·** | S | D | K |
| ZJ01 | · | V | N | **·** | **·** | **·** | **·** | **·** | S | D | K |
| JS-2012 | · | V | N | **·** | **·** | **·** | **·** | **·** | S | D | K |
| LA | · | V | N | **·** | **·** | **·** | **·** | **·** | S | D | K |
| Fa | · | V | N | **·** | **·** | **·** | **·** | **·** | S | D | K |
| Ea | · | V | N | **·** | **·** | **·** | F | **·** | S | D | K |
| SC | · | V | N | **·** | **·** | **·** | **·** | **·** | S | D | K |
| Becker | · | V | N | **·** | V | **·** | **·** | **·** | S | D | K |
| Hercules | T | V | N | **·** | **·** | **·** | **·** | **·** | **·** | · | · |
| Kaplan | · | V | N | **·** | **·** | **·** | **·** | **·** | **·** | · | · |
| Kolchis | T | V | N | **·** | **·** | **·** | **·** | **·** | **·** | · | · |

Continue

| Strain | Amino acid point mutation positions (position of alignment) | | | | | | | | | | |
| --- | --- | --- | --- | --- | --- | --- | --- | --- | --- | --- | --- |
|  | 242 | 273 | 274 | 275 | 276 | 278 | 279 | 280 | 281 | 288 | 294 |
| Bartha | G | P | R | P | R | —— | —— | —— | —— | V | G |
| BP | · | · | · | · | · | · | · | R | P | A | · |
| JY | · | · | · | · | · | · | · | R | P | A | · |
| LGX | · | · | · | · | · | · | · | R | P | A | · |
| MZ1 | · | · | · | · | · | · | · | R | P | A | · |
| MZ2 | D | · | · | · | · | · | · | R | P | A | · |
| NY | · | · | · | · | · | · | · | R | P | A | · |
| SMX | · | · | · | · | · | · | · | R | P | A | · |
| WY | · | · | · | · | · | · | · | R | P | A | · |
| WZ | · | · | · | · | · | · | · | R | P | A | · |
| YY | · | · | · | · | · | · | · | R | P | A | · |
| YZ | · | · | · | · | · | R | P | R | P | A | · |
| ZK | · | · | · | · | · | · | · | R | P | A | · |
| ZM | · | · | · | · | · | · | · | R | P | A | · |
| HN2012 | · | · | · | · | · | · | · | R | P | A | · |
| HNX | · | · | · | · | · | · | · | R | P | A | · |
| HNB | · | · | · | · | · | · | · | R | P | A | · |
| TJ | · | · | · | · | · | · | · | R | P | A | · |
| ZJ01 | · | · | · | · | · | · | · | R | P | A | D |
| JS-2012 | · | · | · | · | · | · | · | R | P | A | · |
| LA | · | —— | —— | —— | —— | · | · | R | P | A | · |
| Fa | · | · | · | · | · | · | · | R | P | A | · |
| Ea | · | · | · | · | · | S | P | R | P | A | · |
| SC | · | · | · | · | · | R | P | R | P | A | · |
| Becker | · | · | · | · | · | · | · | R | P | A | D |
| Hercules | · | · | · | · | · | · | · | · | · | · | · |
| Kaplan | · | · | · | · | · | · | · | · | · | · | · |
| Kolchis | · | · | · | · | · | · | · | · | · | · | · |

Continue

| Strain | Amino acid point mutation positions (position of alignment) | | | | | | | | | |
| --- | --- | --- | --- | --- | --- | --- | --- | --- | --- | --- |
|  | 309 | 303 | 331 | 338 | 342 | 346 | 353 | 384 | 395 | 401 |
| Bartha | H | P | P | A | Q | P | R | K | T | Q |
| BP | R | · | · | V | P | T | · | · | A | · |
| JY | R | · | · | · | P | T | · | · | A | · |
| LGX | R | · | L | · | P | T | · | · | A | · |
| MZ1 | R | · | · | · | P | T | · | · | A | · |
| MZ2 | R | · | · | · | P | T | · | · | A | · |
| NY | R | · | · | V | P | T | · | E | · | L |
| SMX | R | · | · | V | P | T | · | · | A | · |
| WY | R | · | · | V | P | T | · | · | A | · |
| WZ | R | · | · | · | P | T | · | · | A | · |
| YY | R | · | · | · | P | T | · | · | A | · |
| YZ | R | · | · | V | P | T | · | · | A | · |
| ZK | R | · | · | V | P | T | · | · | A | · |
| ZM | R | · | · | · | P | T | C | · | A | · |
| HN2012 | R | · | · | · | P | T | · | · | A | · |
| HNX | R | · | · | · | P | T | · | · | A | · |
| HNB | R | · | · | · | P | T | · | · | A | · |
| TJ | R | · | · | · | P | T | · | · | A | · |
| ZJ01 | R | · | · | · | P | T | · | · | A | · |
| JS-2012 | R | · | · | · | P | T | · | · | A | · |
| LA | R | · | · | · | P | T | · | · | A | · |
| Fa | R | · | · | · | P | T | · | · | A | · |
| Ea | R | · | · | V | P | T | · | · | A | · |
| SC | R | · | · | V | P | T | · | · | A | · |
| Becker | R | · | · | · | · | · | · | · | A | · |
| Hercules | · | · | · | · | · | · | · | · | · | · |
| Kaplan | · | · | · | · | · | · | · | · | · | · |
| Kolchis | · | · | · | · | · | · | · | · | · | · |
